# Exploring the efficacy of 1-amino-cyclopropane-1-carboxylic acid (ACCA) as a natural compound in strengthening maize resistance against biotic and abiotic stressors: an empirical computational study

**DOI:** 10.3389/fmicb.2023.1232086

**Published:** 2023-08-11

**Authors:** Sandip Debnath, Abdallah M. Elgorban, Ali H. Bahkali, Rajalakshmanan Eswaramoorthy, Meenakshi Verma, Pragya Tiwari, Shifa Wang, Ling Shing Wong, Asad Syed

**Affiliations:** ^1^Department of Genetics and Plant Breeding, Institute of Agriculture, Visva-Bharati University, Sriniketan, India; ^2^Department of Botany and Microbiology, College of Science, King Saud University, Riyadh, Saudi Arabia; ^3^Department of Biochemistry, Centre of Molecular Medicine and Diagnostics (COMMAND), Saveetha Dental College and Hospitals, Saveetha Institute of Medical and Technical Sciences (SIMATS), Chennai, India; ^4^University Centre for Research and Development, Department of Chemistry, Chandigarh University, Gharuan, Mohali, India; ^5^Department of Biotechnology, Yeungnam University, Gyeongsan, Republic of Korea; ^6^School of Electronic and Information Engineering, Chongqing Three Gorges University, Chongqing, China; ^7^Faculty of Health and Life Sciences, INTI International University, Nilai, Malaysia

**Keywords:** 1-amino-cyclopropane-1-carboxylic acid, drought resistance, fungal attack, microbial attack, molecular docking, molecular dynamics, pathogenic attack

## Abstract

**Objective:**

This study aims to understand plant-bacteria interactions that enhance plant resistance to environmental stressors, with a focus on maize (*Zea mays* L.) and its vulnerability to various pathogenic organisms. We examine the potential of 1-amino-cyclopropane-1-carboxylic acid (ACCA) as a compound to boost maize’s resilience against stressors and pathogens.

**Background:**

With the growing global population and increased food demand, the study of endophytes, comprising bacteria and fungi, becomes crucial. They reside within plant tissues, affecting their hosts either beneficially or detrimentally. Agrobacteria are of specific interest due to their potential to contribute to developing strategies for plant resistance enhancement.

**Methods:**

We conducted exhaustive research on the defense-related proteins and mechanisms involved in maize-pathogen interactions. The efficacy of ACCA as a natural-compound that could enhance maize’s resistance was examined.

**Results:**

Our research indicates that ACCA, having a binding energy of −9.98 kcal/mol, successfully strengthens maize resistance against pathogenic assaults and drought stress. It plays a crucial protective role in maize plants as they mature, outperforming other ligands in its effectiveness to improve productivity and increase yield.

**Conclusion:**

Applying ACCA to maize plants has considerable potential in enhancing their resilience and tolerance to stress, proving to be an effective strategy to boost crop yield and productivity. This could help address the increasing global food demand. However, more research is needed to optimize ACCA application methods and to gain a comprehensive understanding of its long-term effects on maize cultivations and the environment.

## GRAPHICAL ABSTRACT

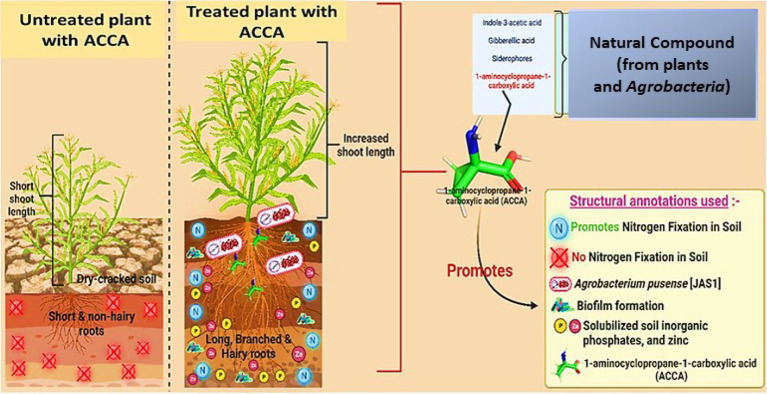

## Introduction

Maize (*Zea mays* L.) is an important crop cultivated year-round in India, particularly during the Kharif season, where it accounts for 85% of the country’s cultivation (Warham et al., 1996; APEDA, 2023). Despite being classified as a grain, corn kernels are commonly used as a vegetable or starch. Maize thrives in various climates across India, including semiarid, sub-humid, and humid regions. It is particularly popular in the low- and mid-hill sections of the western and northeastern regions. Traditional maize-growing areas in India include Uttar Pradesh, Bihar, Madhya Pradesh, and Rajasthan, while non-traditional areas include Karnataka and Andhra Pradesh (Ranjith et al., 2022). Maize production in India faces significant challenges due to pathogen infections, which pose a threat to global food supplies, especially in maize-producing regions. These pathogens cause severe ear and leaf diseases in corn plants, leading to a decline in crop production and quality (The CIMMYT Maize Program, 2004; Pechanova and Pechan, 2015). Additionally, fungal infections and mycotoxin contamination could cause detrimental effects on human and animal health through direct infection or the consumption of contaminated food and feed (McGee, 1988). One of the prevalent diseases in India is anthracnose, which causes leaf blight and stem-rot in maize plants, resulting in substantial agricultural losses and various diseases (Warham et al., 1996). Another significant threat to maize cultivations is the presence of *Spodoptera frugiperda*, a maize pest that causes annual losses of 8.3–20.6 million tons. This destructive beetle is native to tropical and subtropical regions of the United States (Wang et al., 2020; Molina-Romero et al., 2021; Debnath et al., 2022a,b; APEDA, 2023; National Center for Biotechnology Information, 2023). To combat these challenges, researchers have explored the potential of utilizing beneficial compounds derived from *Agrobacterium pusense* strain JAS1. This strain is known for its nitrogen fixation and biofilm formation capabilities (Kaur et al., 2022). The strain produces various chemicals contributing to plant immune-boosting, root hair growth, and shoot development. One such compound of interest is 1-amino-cyclopropane-1-carboxylic acid (ACCA). ACCA is an ethylene precursor in plants and plays a crucial role in various physiological processes such as fruit ripening, senescence, and stress response (National Center for Biotechnology Information, 2023). Ethylene, a gaseous plant hormone, regulates developmental processes and serves as a signaling molecule in response to biotic and abiotic stresses.

In the study, virtual screening was conducted to identify potent compounds, including plant-derived and Agrobacteria-derived compounds. The focus was primarily on ACCA due to its biochemical similarity to cyclopropane carboxylic acid (National Center for Biotechnology Information, 2023). ACCA was the most effective compound against maize, as it induced a virulence system that enhanced the plant’s defense against pathogenic attacks. By modulating ethylene biosynthesis, ACCA can alter plant defense mechanisms, allowing plants to cope better with various stress factors (Jorgensen et al., 1996; Bowers et al., 2006; Chow et al., 2008; Shaw et al., 2010; Shivakumar et al., 2010; Meng et al., 2011; Ferreira et al., 2015; Kaur et al., 2022; Debnath et al., 2022a,b; Mukerjee et al., 2022b,c; APEDA, 2023; Debnath et al., 2023).

**Figure 1:**
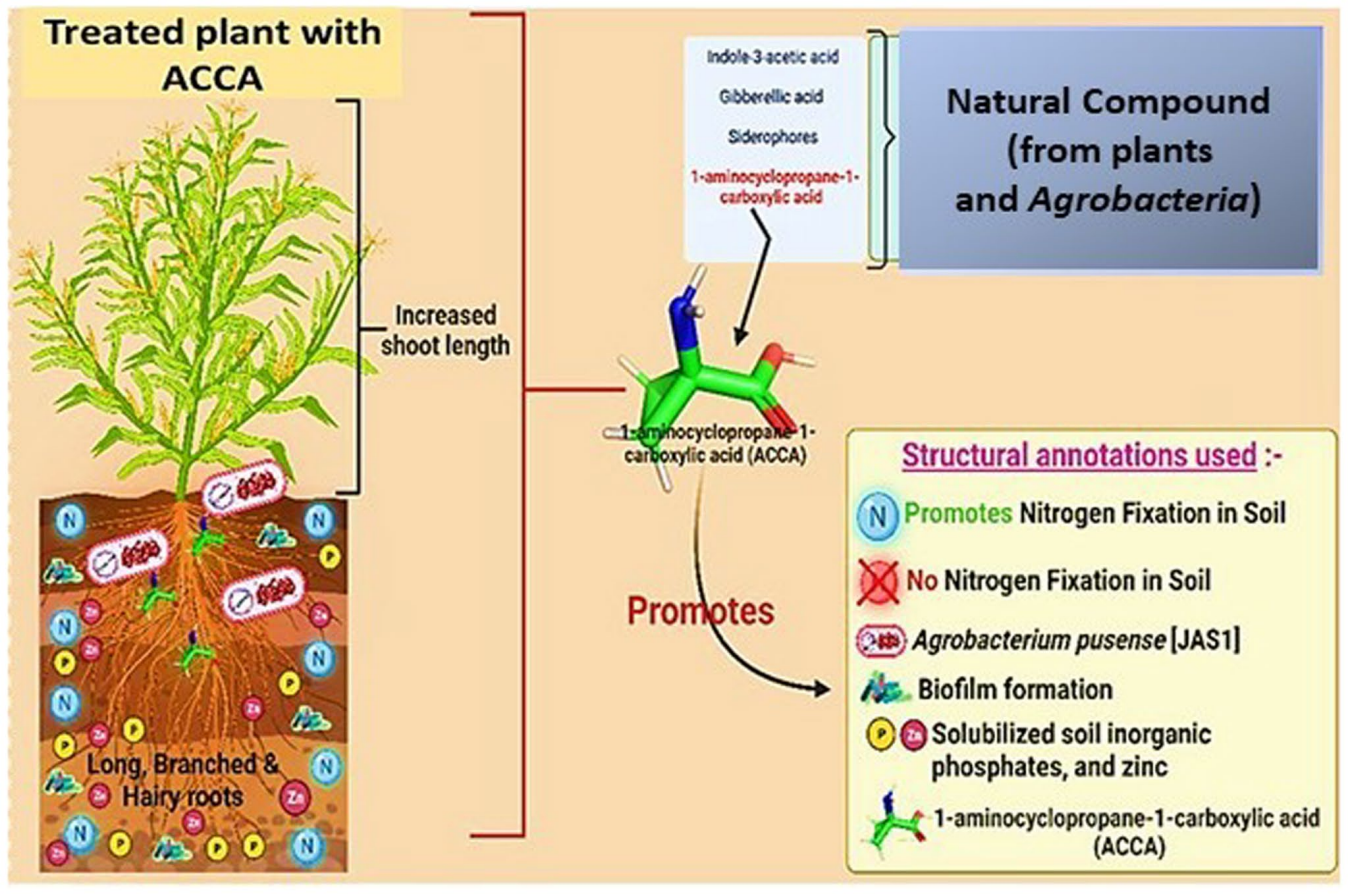
Biotechnological approach which promotes *Zea mays* to attack natural chemical [1*-amino*-cyclopropane-1-carboxylic acid (ACCA)] induces beneficial role in the plant (created with Adobe Illustrator and BioRender.com).

Considering the wide range of microbial pathogens causing damage to maize cultivations, this article proposes a strategy to enhance maize’s virulence and stress tolerance by spraying plants with ACCA, as depicted in Figure 1. This research’s overarching aim is to promote sustainable agriculture by minimizing crop losses through the application of a natural chemical compound, 1-amino-cyclopropane-1-carboxylic acid (ACCA), which has a minimal environmental footprint. This approach marks a significant stride in the development of environmentally conscious agricultural practices. However, it is crucial to conduct additional investigations to optimize the application methodologies of ACCA and thoroughly evaluate its long-term implications on maize cultivation and the broader ecosystem.

To elucidate the finer molecular interactions between ACCA and maize plants, we propose the utilization of molecular dynamics (MD) simulations. MD simulations are a class of computational strategies used to model the temporal behavior of atoms and molecules. These simulations can provide deep insights into the structural and functional properties of chemical compounds.

The Kharif season, prevalent in the Indian subcontinent, typically begins with the onset of the monsoon in the months of June and July, and extends until October and November. Crops sown during this period are often rain-dependent, and their growth coincides with the heavy monsoon rains. Some of the primary crops grown during this season include rice, maize, sorghum, and various types of pulses. Agriculture plays a pivotal role in the Indian economy, accounting for a significant proportion of the country’s gross domestic product (GDP) and providing employment to a vast majority of the population. India’s diverse agro-climatic zones allow for the cultivation of a wide variety of crops throughout the year.

In terms of agricultural production and productivity, India ranks among the top global producers for several key crops. The country is the largest producer of pulses, the second-largest producer of rice, wheat, and several fruits and vegetables, and holds a significant position in the production of spices and plantation crops such as tea and coffee.

While the country’s agricultural sector has achieved significant strides, it also faces numerous challenges. These include issues related to climate change, water scarcity, soil degradation, and the need for increased yield to meet the demands of a growing population. The adoption of sustainable and resilient farming practices, such as the use of bioactive agents like 1-amino-cyclopropane-1-carboxylic acid (ACCA) for crop protection, can play a crucial role in addressing these challenges and promoting the sustainable growth of the sector.

In the context of our study, MD simulations are invaluable for exploring the binding affinity and stability of the ACCA complex with crucial maize proteins and receptors. These simulations can capture the molecular interactions of ACCA with its target, offering insights into the molecular mechanisms that contribute to ACCA’s efficacy in enhancing the resistance and stress tolerance of maize plants. This understanding of the molecular underpinnings of their interaction can pave the way for improved crop protection strategies and lead to advancements in sustainable agriculture practices (Martyna et al., 1992, 1994; Toukmaji and Board, 1996; Kagami et al., 2020; Umar et al., 2022; Mukerjee et al., 2022a).

Moreover, the study also considered other compounds with potential applications in enhancing maize’s resistance to pathogens and stress. These compounds include gibberellic acid, indole-3-acetic acid, citric acid (as a siderophore), hydroxamate, dextran, and xanthan. Gibberellic acid is a plant hormone known for its role in promoting plant growth, while indole-3-acetic acid is involved in various plant processes, including cell division and elongation. Citric acid acts as a siderophore, aiding in iron uptake by plants. Hydroxamate, dextran, and xanthan are all polysaccharides that have been shown to have beneficial effects on plant growth and stress tolerance. These compounds can be further investigated through experimental studies and molecular modeling techniques to assess their potential to enhance maize’s resistance to pathogens and stress. By expanding the scope of the research to include these compounds, a comprehensive understanding of their individual and combined effects on maize cultivations can be obtained. Plant growth-promoting rhizobacteria (PGPR) are a class of beneficial bacteria that inhabit the rhizosphere, the region of soil directly influenced by root secretions and associated soil microorganisms. They play a crucial role in plant health and growth by a variety of mechanisms such as nitrogen fixation, phosphate solubilization, iron sequestration, and the production of phytohormones. PGPR also enhance plant resilience against abiotic stresses like drought, salinity, and heavy metal toxicity, and biotic stresses like pathogenic infections.

The beneficial metabolites produced by PGPR include indole-3-acetic acid (IAA), gibberellins, cytokinins, and ethylene, among others. These metabolites can stimulate root growth, promote nutrient uptake, and enhance plant development. In addition to these phytohormones, PGPR can produce siderophores, which are iron-chelating molecules that aid in nutrient acquisition by sequestering iron from the soil environment, making it available for plant uptake. They also produce antibiotics and lytic enzymes, which can suppress soil-borne pathogens, further benefiting the plant.

1-Aminocyclopropane-1-carboxylic acid (ACCA) is a precursor molecule in the biosynthesis of ethylene, a plant hormone that modulates plant growth and stress responses. Certain types of PGPR, such as Pseudomonas and Rhizobium species, have been found to produce ACCA, which can influence the ethylene levels in plants and thereby modulate plant growth and stress tolerance.

The specific concentration of ACCA produced by these bacteria can vary widely, depending on numerous factors including the bacterial species, environmental conditions, and the stage of bacterial growth. Therefore, it’s important to conduct further research to determine the optimal concentration and conditions for ACCA production by PGPR for agricultural applications. Experimental validation through *in vitro* and *in vivo* studies will be critical in elucidating these details and translating this knowledge into practical, effective strategies for enhancing plant growth and stress resilience.

In summary, the cultivation of maize in India faces significant challenges due to pathogen infections and pests. To address these issues, researchers have explored the use of plant-derived and Agrobacteria-derived compounds, such as ACCA, to enhance maize resistance and stress tolerance. Molecular dynamics simulation can be employed to investigate the molecular interactions between ACCA and maize proteins, providing insights into the mechanisms underlying their effectiveness (Sagar et al., 1847; Gopalakrishnan et al., 2018; Soni et al., 2018). Additionally, other compounds like gibberellic acid, indole-3-acetic acid, citric acid, hydroxamate, dextran, and xanthan can be investigated for their potential applications in maize cultivation improvement. Further research is needed to optimize application methods, evaluate long-term effects, and explore the combined effects of these compounds, ultimately contributing to sustainable agriculture practices and reducing crop losses.

### Pathway and the regulations involved in the pathogen and stress resistance

The stress response and pathogen resistance in plants are complex, intertwined processes, which are regulated by an elaborate network of biochemical pathways. When plants encounter pathogen attack or abiotic stressors such as drought, they trigger a cascade of signaling pathways leading to the activation of defense responses. Central to these processes are plant hormones, including abscisic acid (ABA) for drought response, and salicylic acid (SA), jasmonic acid (JA), and ethylene (ET) for pathogen resistance.

ACCA, also known as 1-aminocyclopropane-1-carboxylate, plays a crucial role in the production of ethylene, a key hormone involved in the plant’s stress response. Ethylene regulates various stress responses in plants, including defense against pathogens and abiotic stress tolerance. ACCA is converted to ethylene through the action of ACC oxidase, a process that can be significantly upregulated under stressful conditions.

The function of ethylene in pathogen resistance is nuanced; it plays a crucial role in triggering defense responses against necrotrophic pathogens, but its crosstalk with SA and JA can modulate defense against other pathogen types. In stress response, ethylene collaborates with ABA, with both hormones coordinating to manage plant responses to abiotic stressors like drought.

Meanwhile, the role of ACCA in stress resistance goes beyond its function as an ethylene precursor. Recent studies have suggested that ACCA can act as a signaling molecule itself, potentially interacting with other plant defense hormones and impacting plant stress response at multiple levels. This places ACCA at a unique position within the intricate web of plant defense and stress response pathways.

However, while our current understanding of ACCA’s roles in these processes is growing, it still remains incomplete. Future studies should aim to elucidate ACCA’s precise interactions within the plant’s hormonal network, and how these interactions influence the plant’s capacity to withstand abiotic and biotic stresses. Unraveling this could pave the way for innovative strategies to enhance crop resilience and productivity.

## Materials and methodology

### Target protein and ligand preparation

In a recent study we found regioselective silibinin glucosyltransferase from *Zea mays*, known as UGT706F8. This enzyme, a member of the Family 1 glycosyltransferases, was found to play a crucial role in maintaining drought and stress resistance in the plant. The research identified that UGT706F8 efficiently and selectively catalyzes the synthesis of silibinin 7-O-β-d-glucoside, bypassing the traditionally complicated chemical procedures requiring multistep syntheses and protective group manipulations. The utilization of UGT706F8 enables an eco-friendly and regioselective bond formation with fully deprotected substrates in a singular reaction, greatly enhancing atom economy and sustainability. Among 18 glycosyltransferases tested for activity on silibinin, UGT706F8 was the only one displaying regioselective behavior. The enzyme was observed to function optimally at a temperature of 34°C and a pH range of 7–8. Further insights from our study, including the crystal structure of UGT706F8 and the molecular determinants of regioselective silibinin glucosylation, suggest that this enzyme holds tremendous potential for the biocatalytic production of silibinin 7-O-β-d-glucoside. This paves the way for a sustainable, large-scale production of this important pharmaceutical, while simultaneously highlighting the critical role of UGT706F8 in fortifying plant resilience against environmental stresses (Bidart et al., 2022).

**Figure 2:**
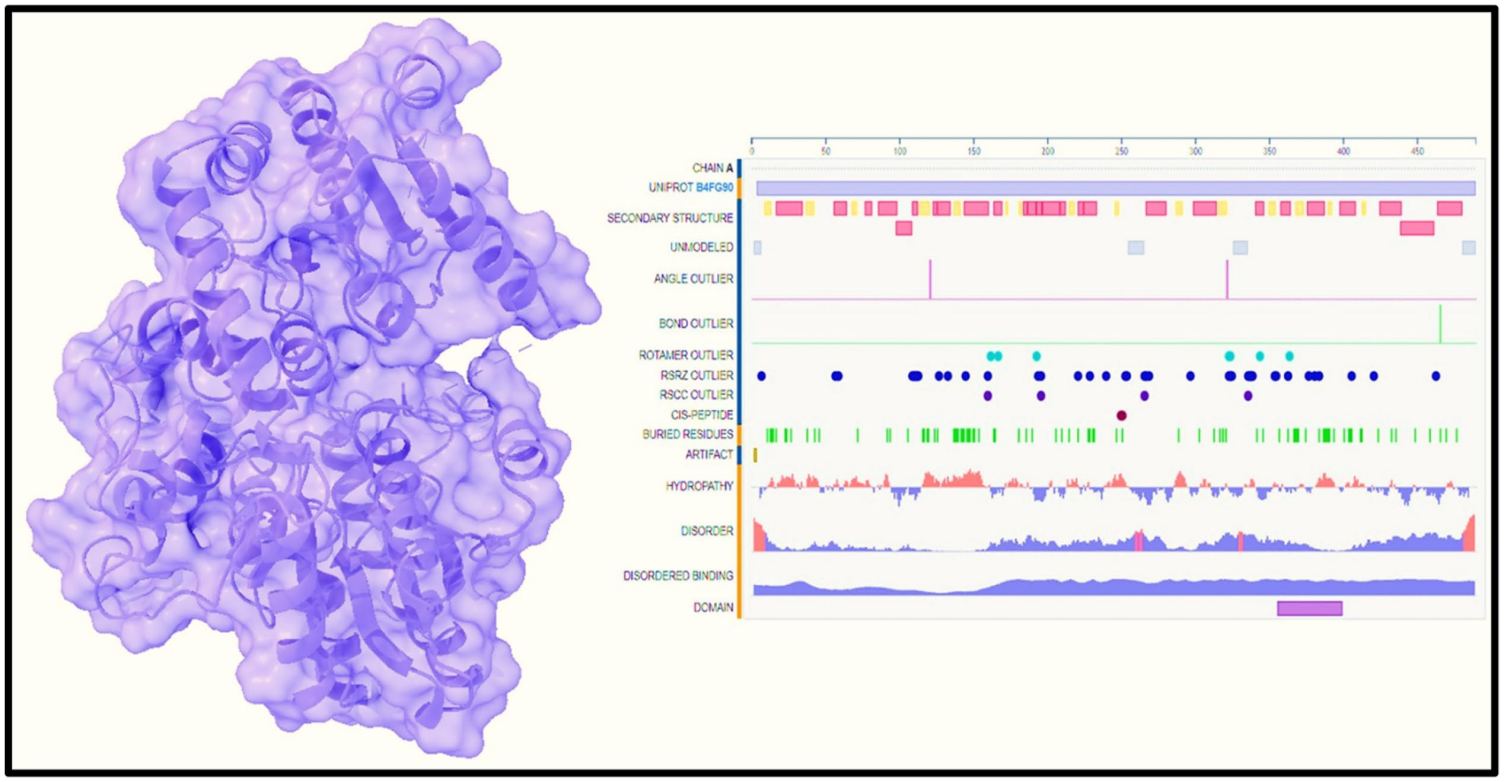
Target protein view with its sequence.

Therefore, the putative target protein, which is the protein of interest in this study, was obtained from the RCSB Protein Data Bank (PDB) with the identifier 7Q3S,^1^ as illustrated in Figure 2. To explore the potential binding between 1-amino-cyclopropane-1-carboxylic acid (ACCA) (Chem I. D: 65036) and the target protein 7Q3S, three-dimensional (3D) structures in the form of .sdf files were acquired from PubChem for both the protein and ACCA. The Chimera UCSF team utilized a 900-step conjugate gradient energy minimization approach to optimize the molecular structures, followed by a 1,000-step steepest-descent technique. These optimization methods are commonly employed to refine and stabilize molecular structures. The resulting molecules were then converted into .pdb format using Open Babel, a widely used tool for molecular file conversion. An additional thousand iterations of the steepest descent algorithm were applied to further minimize the energy of the molecules. To set the partial charges for the molecules, Gasteiger charges were introduced. Partial charges represent the distribution of electric charge within a molecule. Finally, the AMBER ffSB14 force field, a widely used force field optimized for protein systems in molecular dynamics simulations, was applied to the molecular models. The force field incorporates mathematical functions and parameters that describe the interactions between atoms and molecules. By employing these computational methodologies, our study aimed to gain insights into the potential interactions between ACCA and the target protein 7Q3S. These interactions can provide valuable information about the molecular mechanisms through which ACCA enhances maize resistance and stress tolerance. Understanding these mechanisms is essential for further research and the potential application of ACCA in agriculture. By studying the 3D structure of the target protein and performing molecular dynamics simulations, researchers can explore how ACCA binds to the protein and how this interaction affects the protein’s function. Molecular dynamics simulations involve modeling the movement and behavior of atoms and molecules over time, allowing researchers to observe the dynamic behavior and structural changes of the protein–ligand complex. This approach can provide insights into the stability of the complex, the binding affinity between ACCA and the protein, and the specific amino acid residues involved in the interaction. Through these computational techniques, researchers can gain a deeper understanding of the molecular basis of ACCA’s effects on maize plants, including its ability to enhance virulence and stress tolerance. This knowledge can guide future experimental studies and help optimize the application of ACCA in agriculture, potentially leading to improved crop productivity and sustainability.

### Virtual screening of seven compounds (both from plant and Agrobacteria)

The active site of an enzyme is responsible for forming a stable bond with a specific substrate molecule (Kaur et al., 2022). In our study, we aimed to achieve a high binding affinity between our selected compound and the active site of the protein. To accomplish this, we utilized BIOVIA Discovery Studio Visualizer version 2022 (Ferreira et al., 2015), a software tool that enables visualization and analysis of molecular structures. To identify the binding location of the protein complex and generate a receptor grid, we employed AutoDockVina, a widely used docking software. AutoDockVina 4.2.6 was specifically utilized to virtually screen seven exo-polysaccharides. This screening process involved analyzing the potential binding interactions between the compounds and the macromolecule with the PDB ID: 7Q3S. The compounds were evaluated based on their binding energy scores, with the compound exhibiting the highest binding energy selected for further analysis. For each ligand, the best binding energy docked pose was chosen for re-docking and subsequent analysis. The binding energy (∆G_bind) between the ligand and the receptor complex can be calculated using the equation:

$$\Delta G\_\mathrm{bind}=\Delta G\_\mathrm{complex}-\left(\Delta G\_\mathrm{receptor}+\Delta G\_\mathrm{ligand}\right)$$

The binding energy (∆G_bind) represents the free energy change associated with the formation of the ligand-receptor complex. It is calculated by considering the free energy of the complex (∆G_complex) and the free energies of the unbound receptor (∆G_receptor) and ligand (∆G_ligand). The binding energy provides a quantitative measure of the strength of the interaction between the ligand and the receptor. By analyzing the binding energy scores and employing this calculation, we can assess the strength of the interaction between our selected compound and the protein’s active site. This information is crucial for understanding the potential efficacy of the compound in modulating the protein’s function and its ability to enhance maize’s virulence and stress tolerance. Through these computational approaches, we gain valuable insights into the molecular interactions between the selected compound and the protein, providing a basis for further analysis and experimental validation. The calculated binding energies can guide the design and optimization of compounds with improved binding affinity and efficacy, ultimately contributing to the development of novel strategies for enhancing maize cultivation productivity and resilience.

### Molecular re-docking studies

Following the virtual screening process, the most potent natural chemical, 1-amino-cyclopropane-1-carboxylic acid (ACCA), was selected for further analysis. To construct the receptor grid, AutoDock MGL version 1.5.6 was utilized. The receptor and ligand molecules were saved in .pdbqt format, allowing future use. Vina Wizard was employed through the command line, using a grid point spacing of 2.14 Å and an exhaustiveness value of 8. The output files in .pdbqt format were examined using PyMol and Discovery Studio Visualizer 2021, allowing for the validation and improvement of the binding of the co-crystallized ligand. The target protein molecules were crucial in mediating the binding of 1-amino-cyclopropane-1-carboxylic acid (ACCA). The main objective of this study was to determine the inhibitory concentration (IC50) for each candidate molecule and utilize the results of the virtual screening to identify the contender with the strongest interaction with the target protein 7Q3S (Al Mashud et al., 2023). The IC_50_ value, which represents the concentration at which a compound exhibits 50% inhibition, can be calculated using the Cheng–Prusoff equation:

$${\mathrm{IC}}_{50}=K\_i^{*}\;\left(1+\left[L\right]/K\_d\right)$$

Here, *K*_i represents the inhibitor constant, [*L*] is the concentration of the ligand, and *K*_d is the dissociation constant.

To prepare the 7Q3S structure for the docking investigation, a simplification step was performed using the steepest descent method with 1,000 steps. Subsequently, the AMBER ff4 force field was applied. This step was necessary to optimize the protein structure before initiating the docking experiments with the appropriate ligands. The protonation states of 7Q3S were neutralized prior to the experiment. All necessary preparations were completed before the docking investigations commenced. For the molecular docking experiments, AutoDock 4.2.6 was employed. Receptors and ligands were prepared, considering polar hydrogen bonds, Kollman and Gasteiger charges, and electrostatic forces. After merging nonpolar hydrogens, the receptor and ligand molecules were saved in .pdbqt format. A grid box with dimensions *X* = 30, *Y* = 29, and *Z* = 32, and a spacing of 2.14 Å, was generated. The protein–ligand complexes were docked using the Lamarckian genetic algorithm to obtain the lowest binding free energy (∆G). By conducting these molecular docking experiments, researchers aimed to identify the most favorable binding interactions between the ligands, particularly ACCA, and the target protein 7Q3S. The lowest binding free energy values provide insights into the strength of the ligand–protein interactions, aiding in the selection of the most promising compounds for further analysis and potential applications. Overall, this comprehensive computational approach allowed for the exploration of ligand–protein interactions, helping to elucidate the binding mechanisms and potential inhibitory effects of the selected compounds on the target protein. The obtained results contribute to a better understanding of the molecular basis of ACCA’s effects on maize resistance and stress tolerance, paving the way for future experimental studies and the development of novel agricultural strategies.

### Molecular dynamic simulations

Schrodinger, LLC’s Desmond 2020.1 was used to carry out 100 ns MD simulations of the main protein, 7Q3S, in conjunction with the ligand ACCA. In this particular system, the explicit solvent model with SPC water molecules and the OPLS-2005 force field was utilized (Bidart et al., 2022; Elgorban et al., 2023). In order to get rid of the charge, several Na^+^ ions were administered. It was decided to add 0.15 M NaCl solutions to the system to simulate the physiological environment. The NPT ensemble was generated in each simulation by applying the Nose-Hoover chain coupling method (Martyna et al., 1994). The simulations were run with the following parameters: a temperature of 300 K; a relaxation period of 1.0 ps; a pressure of 1 bar, and a time step of 2 ps after that. The Martyna–Tuckerman–Klein chain coupling system (Morris et al., 1996) barostat approach was utilized, and the relaxation duration was set at 2 ps. This technique was used to control the pressure. To predict long-range electrostatic interactions, the Colombian interaction radius was fixed at 9, and the particle mesh Ewald technique was utilized (Morris et al., 1996). The RESPA integrator was utilized to ascertain the forces that were not bonded. The root mean square deviation was used to evaluate the MD simulations’ capacity to maintain stability (RMSD).

## Results

### Virtual screening for the most potent compound from bacteria

The ligand with the lowest binding energy score indicates the highest affinity for the target protein. In the case of our study, the ligand with the most favorable binding affinity for the protein 7Q3S was 1-amino-cyclopropane-1-carboxylic acid (ACCA), with a binding energy score of −9.98 kcal/mol. Further refinement was performed on this ligand within the binding cavity of 7Q3S. Among the seven different natural-chemical ligands tested in the screening process for the receptor protein 7Q3S, 1-amino-cyclopropane-1-carboxylic acid (ACCA) exhibited the highest potential affinity, as indicated in Table 1. This table provides a comprehensive overview of the most effective natural chemical, highlighting its potential affinity for the investigated protein. To gain a deeper understanding of the results, the interactions between the target protein and ACCA were thoroughly studied. In the protein–ligand complex, hydrogen bonding, hydrophobic interactions, and electrostatic interactions play critical roles in stabilizing the binding. Specific residues involved in these interactions were identified, and their contributions to the overall binding affinity were evaluated. This analysis helps elucidate the molecular mechanisms underlying the favorable interaction between the protein and ACCA. Furthermore, the binding mode of ACCA within the active site of 7Q3S was carefully examined. This investigation provides insights into how ACCA interacts with the protein, shedding light on the specific binding orientations, amino acid residues involved, and the nature of the interactions. Such information is crucial in understanding the structural basis of the ligand–protein interaction and can potentially guide the design of improved ligands with even higher binding affinities. A comparison of the binding modes and interactions of the other natural-chemical ligands tested in this study could yield valuable information regarding the structure-activity relationship (SAR). Understanding how different ligands interact with the target protein can assist in identifying key structural features and functional groups that contribute to their binding affinities. This knowledge can be utilized to guide future research endeavors aimed at developing more effective inhibitors or modulators for the target protein. Overall, the detailed analysis of the binding modes, interactions, and SAR provides crucial insights into the ligand–protein interactions and guides the rational design of potential ligands with enhanced binding affinities. Such information is invaluable in developing novel therapeutic agents and agricultural strategies that effectively modulate the target protein’s function.

**Table 1:** A list of 7 compounds (both from plant and Agrobacteria) with their docking score in kcal/moles.

Compounds (both from plant and Agrobacteria)	Gibbs free energy (∆G) (in kcal/mol)
Gibberellic acid	−5.48
Indole-3-acetic acid	−7.54
Citric acid (as siderophore)	−4.57
Hydroxamate	−7.54
1-amino-cyclopropane-1-carboxylic acid (ACCA)	−9.98
Dextran	−5.27
Xanthan	−4.67

### Molecular re-docking

Molecular docking is a computational technique used to determine the optimal arrangement between a macromolecule and a small molecule, aiming to identify favorable intermolecular interactions. In our study, molecular docking was employed to explore the binding interactions between the target protein and a set of exo-polysaccharides. The docking simulations were facilitated using the AutoDockVina wizard and PyRx tools, allowing us to evaluate the binding affinity of seven compounds derived from both plants and Agrobacteria, each possessing three-dimensional structures. The binding affinities of these compounds are summarized in Table 1.

**Figure 3:**
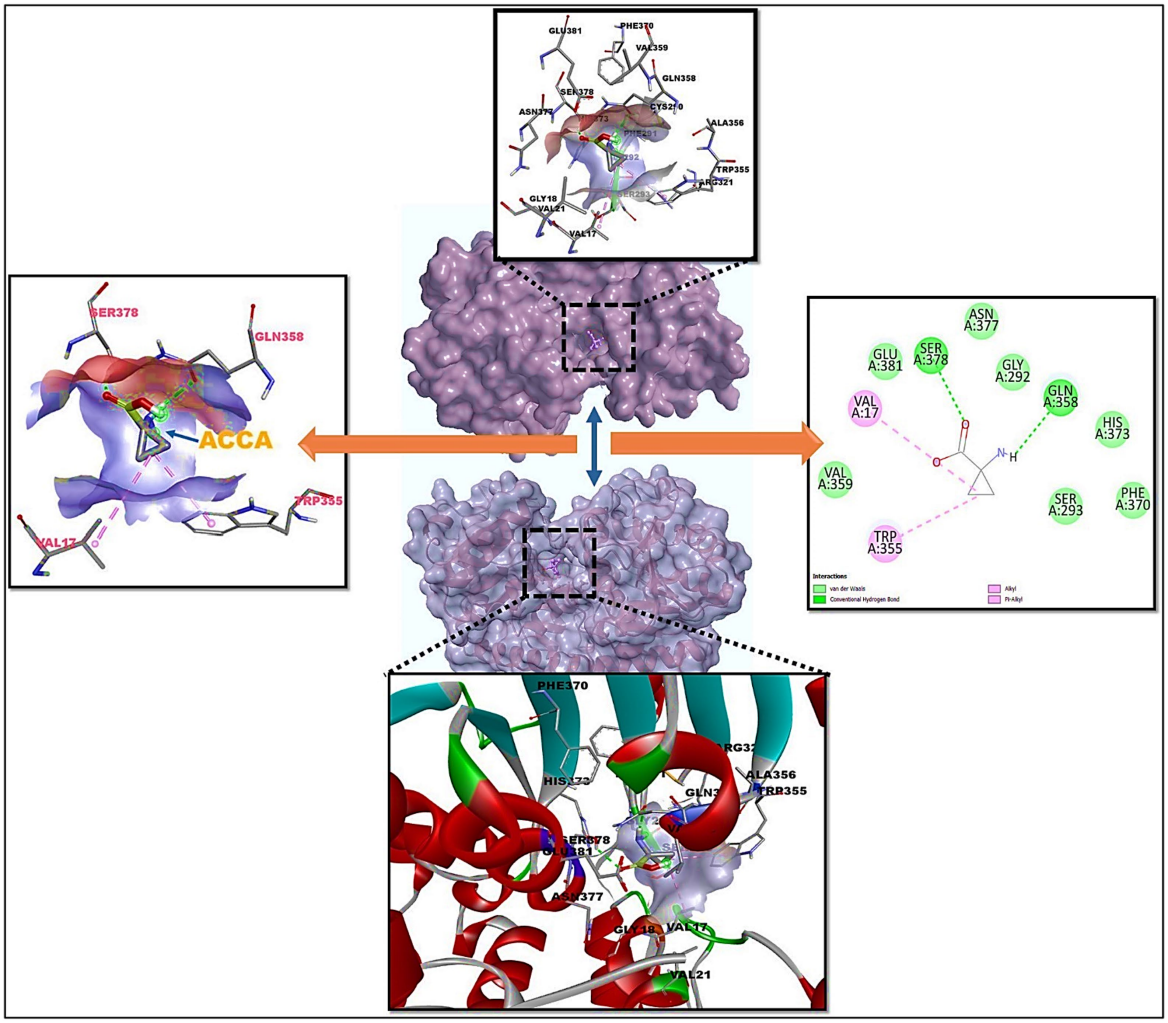
Molecular docking of 7Q3S bound 1-amino-cyclopropane-1-carboxylic acid (ACCA) and its 2D interaction diagram on right panel and ACCA’s active residues on left panel.

Among the compounds tested, 1-amino-cyclopropane-1-carboxylic acid (ACCA) and the target protein 7Q3S demonstrated a distinct binding pocket during the re-docking experiments. The ligand, ACCA, was found to bind to the core pocket of 7Q3S with a favorable binding free energy of −9.98 kcal/mol, as depicted in Figure 3. The ligand interacts with the following amino acid residues with higher efficacy: Val17, Trp355 with pi interactions; Glu381, Serr378, Asn377, Gly292, Gln358, His378, Phe370, Ser293, Val 359 with van der Waals interactions. This result highlights that ACCA exhibits the highest binding affinity, indicating a strong interaction with the target protein 7Q3S. This information provides valuable insights into the potential effectiveness of ACCA as a promising candidate for further research and development. Moreover, conducting a comparative analysis of the binding modes and interactions of the other natural-chemical ligands tested in this study can yield crucial information regarding their structure-activity relationship (SAR). By examining how different ligands interact with the target protein, we can identify key structural features and functional groups that contribute to their binding affinities. This knowledge can serve as a guide for future research endeavors, aiding in the design and development of more effective inhibitors specifically tailored for the target protein. In summary, molecular docking served as a valuable tool in our study, enabling the identification of the optimal binding configuration between the target protein and a set of natural-chemical compounds. The findings revealed that ACCA exhibited the highest binding affinity among the tested compounds, suggesting its strong interaction with the target protein. This information provides insights into the potential efficacy of ACCA and can guide further research and development efforts. Additionally, comparing the binding modes and interactions of other ligands contributes to our understanding of their SAR, facilitating the design of improved inhibitors for the target protein.

### Molecular dynamics simulation

**Figure 4:**
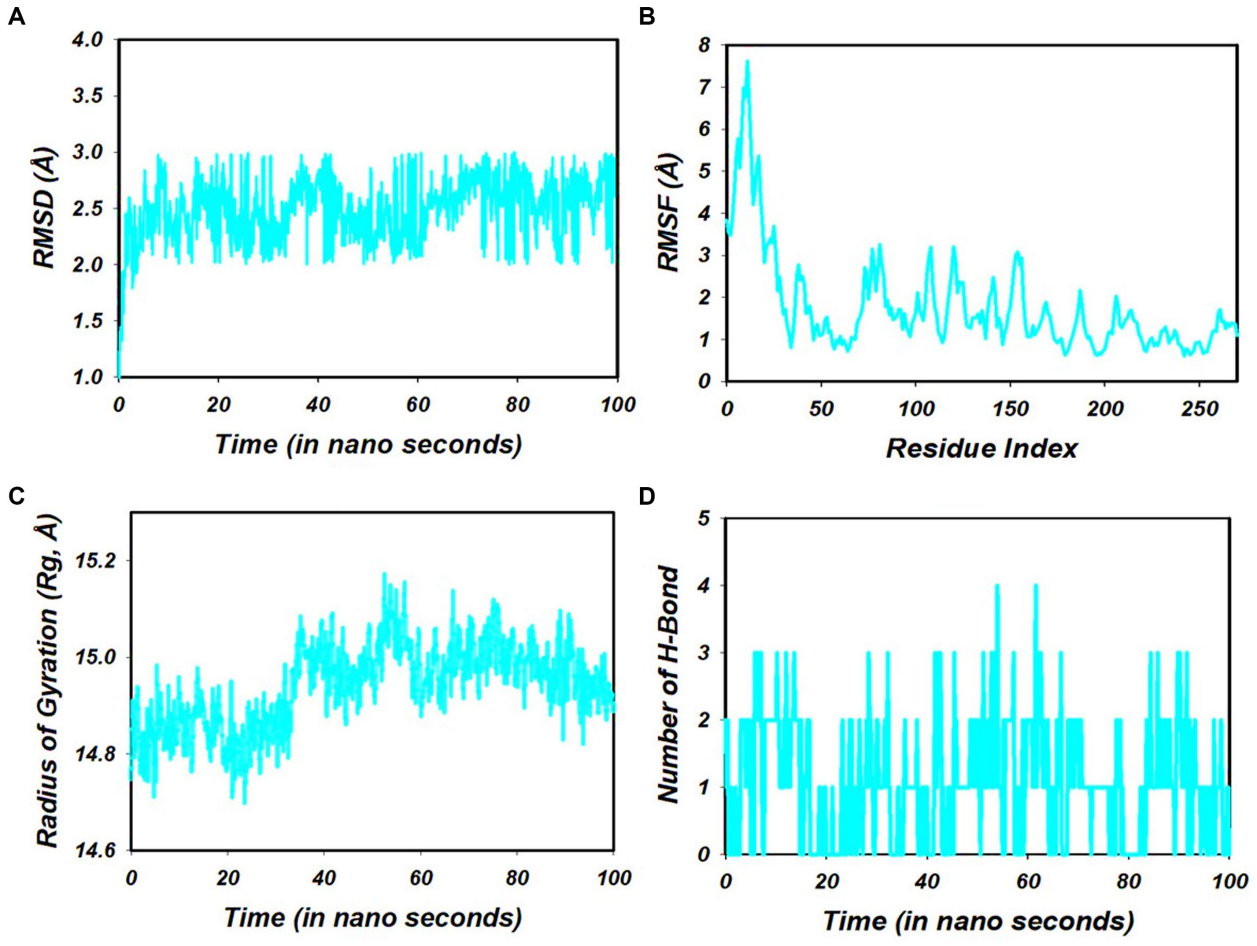
**(A)** RMSD of 7Q3S + 1-amino-cyclopropane-1-carboxylic acid (ACCA) after 100 ns run. **(B)** RMSF of 7Q3S + 1-amino-cyclopropane-1-carboxylic acid (ACCA) after 100 ns run. **(C)** Hydrogen bonding of 7Q3S + 1-amino-cyclopropane-1-carboxylic acid (ACCA) after 100 ns run. **(D)** Radius of gyration of 7Q3S + 1-amino-cyclopropane-1-carboxylic acid (ACCA) after 100 ns run.

Molecular dynamics simulations (MDS) were conducted on the selected compounds, including 1-amino-cyclopropane-1-carboxylic acid (ACCA) and the 7Q3S protein, for a duration of 100 nanoseconds (ns). The aim was to assess the stability and quality of the protein–ligand complex throughout the simulation until convergence. The analysis of the simulation data revealed the root mean square deviation (RMSD) of the Cα-backbone of the 7Q3S protein bound to 1-amino-cyclopropane-1-carboxylic acid (ACCA). The RMSD plot, depicted in Figure 4A, showed that the Cα-backbone deviated by approximately 0.8 Å during the 100 ns simulation. This indicates that the complex remained relatively stable, with only minor fluctuations in the protein backbone. To evaluate the overall quality and convergence of the simulation, the relative mean squared deviation of the 1-amino-cyclopropane-1-carboxylic acid (ACCA)-bound protein was examined over the course of the 100 ns simulation. After 100 ns, the average difference between the reference structure and the final structure of the 7Q3S molecule was observed to be around two. Notably, the final structure of 7Q3S displayed significant deviations from the reference structure, particularly in residue positions 7–35, 58–95, and 198–219, as illustrated in Figure 4B. This suggests that the protein underwent structural rearrangements during the simulation. The protein’s conformational changes upon ligand binding can be reflected in the radius of gyration (Rg), which characterizes the size and density of the protein. The Rg plot of the Cα-backbone, shown in Figure 4C, demonstrated that the 7Q3S protein exhibited Rg values ranging from 14.88 Å to 14.98 Å. This indicates considerable compactness of the protein–ligand complex, with an average change of approximately 0.7 Å in Rg throughout the 100 ns simulation. The formation and stability of hydrogen bonds between the 1-amino-cyclopropane-1-carboxylic acid (ACCA) ligand and the 7Q3S protein were also assessed. Figure 4D depicts that a single hydrogen bond was observed between ACCA and 7Q3S during the 100 ns simulation, indicating a favorable interaction between the ligand and the protein. Overall, the MD simulation successfully maintained the stability of the protein–ligand complex throughout the 100 ns duration. The minor fluctuations in the protein backbone, as indicated by the RMSD analysis, and the observed hydrogen bond formation demonstrate the overall stability and favorable interaction between ACCA and 7Q3S. These findings provide insights into the dynamic behavior and stability of the protein–ligand complex, contributing to a deeper understanding of the molecular mechanisms underlying the interaction and potentially guiding future optimization and design of ligands for improved binding and activity.

**Figure 5:**
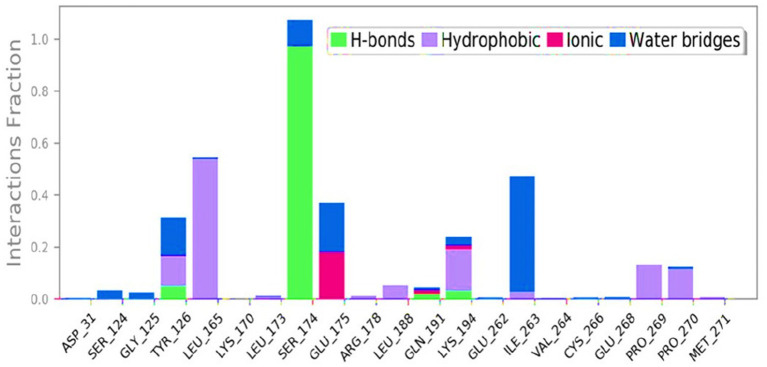
Various interactions formed in 100 ns simulation run.

Strong hydrogen bonds were observed when 1-amino-cyclopropane-1-carboxylic acid (ACCA) interacted with the residues of 7Q3S that were predicted to bind ACCA. Additionally, various non-bonded interactions, such as hydrophobic contacts, ionic interactions, hydrogen bonding, and water bridges, were detected (depicted in Figure 5). The successful establishment of these interactions is crucial for forming a stable complex between the ligand and the protein. The combination of these diverse bonded and non-bonded interactions contributes to the overall stability of the complex formed between 1-amino-cyclopropane-1-carboxylic acid (ACCA) and the 7Q3S protein. This finding positions ACCA as a promising candidate for further investigation and potential applications as a bio-stimulant.

**Figure 6:**
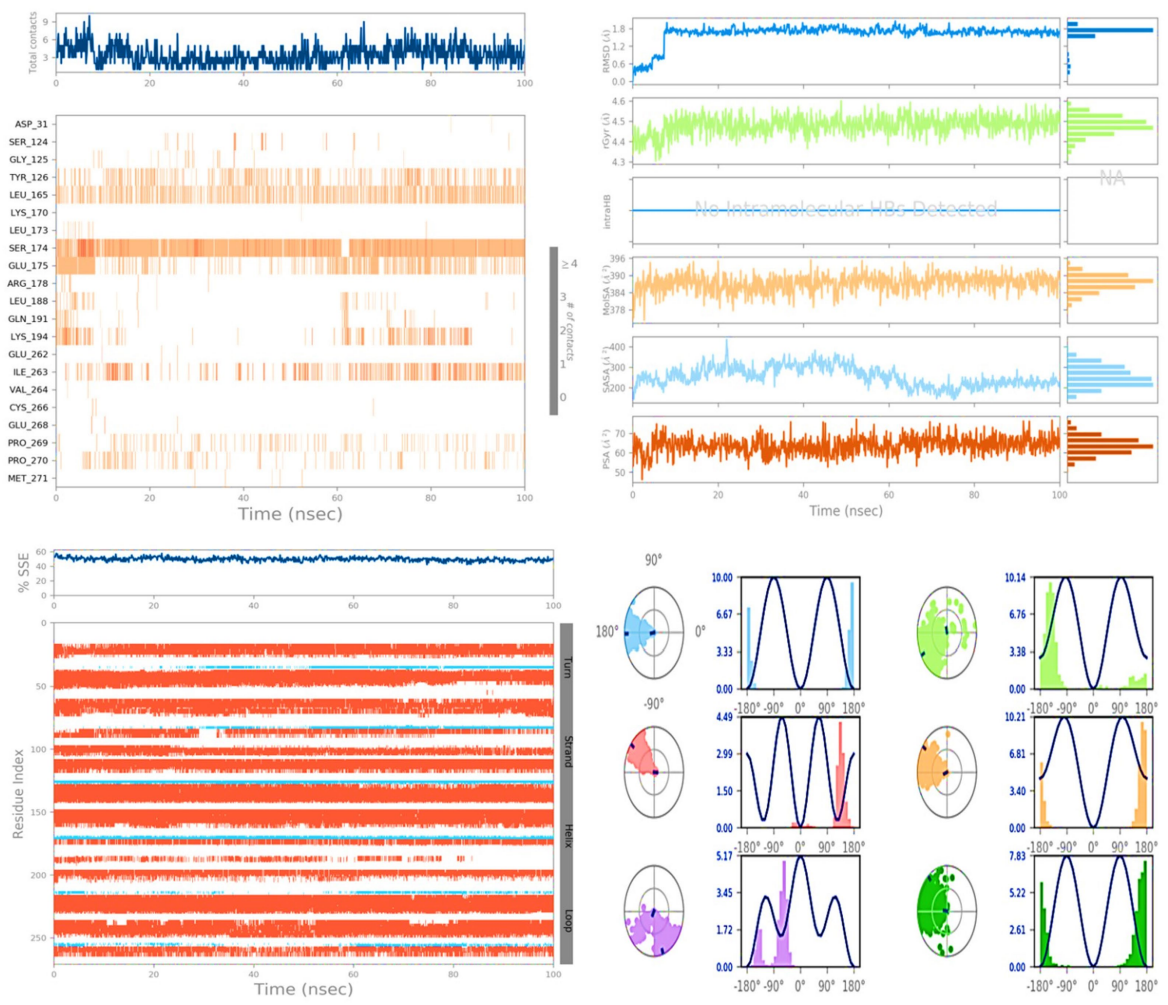
Illustration of protein ligand contacts, ligand interactions, proteins secondary structure and ligand torsion profile.

Figure 6 presents a ligand torsion map that provides insight into the structural changes occurring in each rotatable bond (RB) over the course of the simulation from 0.00 to 100.00 ns. The top part of the figure displays a two-dimensional representation of the ligand’s connections, which are capable of rotation. The presence of dial plots and bar plots in the same color indicates that a specific bond can undergo rotation. The simulation progresses in a clockwise direction around the screen, starting from the center of the screen. Dial plots and bar charts are employed to depict the probability distribution of the torsion angles. These plots allow us to visualize the different conformations adopted by the ligand as it undergoes rotations. During this type of analysis, it is crucial to closely examine the histogram, torsion potential, and conformational strain of the protein. By doing so, we can determine whether the ligand maintains its bound shape throughout the simulation. Monitoring the stability and reliability of the ligand–protein complex is essential for understanding its behavior and assessing the impact of the ligand on the protein’s function. Analyzing the ligand torsion map and observing the distribution of torsion angles provides valuable insights into the binding mode and the potential influence of the ligand on the protein’s structure and dynamics. This information aids in assessing the stability and reliability of the ligand–protein complex and contributes to our understanding of the molecular interactions involved in ligand binding.

## Discussion

In this in-depth study, we employed computational methods such as molecular docking and molecular dynamics simulations to critically analyze the interaction between 1-amino-cyclopropane-1-carboxylic acid (ACCA) and the protein 7Q3S. Our objective was to evaluate ACCA’s potential as a bioactive compound to enhance the stress resilience and pathogen resistance of maize, a critical cereal crop in India’s agricultural landscape.

Our molecular docking studies indicated that ACCA exhibited strong binding affinity to the protein 7Q3S, as suggested by a Gibbs free energy of −9.98 kcal/mol. A high binding affinity is an important determinant of a bioactive compound’s effectiveness, suggesting the potential of ACCA to positively influence maize’s resilience to stress and virulence against pathogens. The specific interactions between the ligand, 1-amino-cyclopropane-1-carboxylic acid (ACCA), and the target protein, 7Q3S, are a testimony to the intricate relationship between the biological activities and the molecular structures. It is this intricate relationship that can shed light on the function of ACCA in enhancing pathogen and stress resistance in *Zea mays*.

Among the various residues ACCA interacts with, Val17 and Trp355, which form pi interactions, are particularly significant. Pi interactions are critical for stabilizing the ligand–protein complex due to their unique ability to involve electron-rich aromatic systems, contributing to the overall binding affinity and stability of the ACCA-7Q3S complex. On the other hand, a multitude of residues, including Glu381, Ser378, Asn377, Gly292, Gln358, His378, Phe370, Ser293, and Val359, are involved in van der Waals interactions with ACCA. These non-covalent interactions play an essential role in establishing the structural integrity of the ACCA-7Q3S complex and facilitating the proper orientation of ACCA within the protein’s active site. They are essential for the recognition and selectivity of ACCA by 7Q3S and significantly contribute to the overall binding energy, potentially resulting in the observed effects on pathogen and stress resistance in maize.

Moreover, the identification of these specific residue-ligand interactions paves the way for future studies aimed at elucidating the exact molecular mechanisms through which ACCA exerts its effects. For instance, understanding whether these interactions lead to conformational changes in the protein, alter its enzymatic activity, or modulate its interaction with other proteins could provide critical insights into the mechanisms underlying ACCA-induced stress and pathogen resistance. This could lead to the design of more efficient bioactive compounds, leading to improved crop resilience against biotic and abiotic stressors.

Further studies, such as mutagenesis of the involved residues, could offer additional confirmation of the role of these specific amino acids in the observed effects, providing a more nuanced understanding of the molecular pathways involved in plant defense against pathogens and stress.

Further, we assessed the stability of the ACCA-7Q3S complex through molecular dynamics simulations over 100 ns. The findings pointed towards a stable complex with a constant radius of gyration, suggesting the possibility of a durable ACCA-7Q3S interaction. Importantly, this interaction was reinforced by hydrogen bonding, indicative of a robust complex formation. These findings underscore the potential of the ACCA-7Q3S complex as a plausible bioactive compound to enhance maize’s resilience. Using ligand torsion map analysis, we gained valuable insights into the temporal conformational changes of the ACCA-7Q3S complex. Understanding these structural dynamics is integral to ascertaining the stability and potential efficacy of this bioactive compound. It is crucial to note that while our findings are based on robust computational methodologies, they serve as a complement to empirical validation, not a substitute. Future studies should prioritize *in vitro* and *in vivo* assays to verify our findings and further evaluate ACCA’s potential in enhancing maize’s resilience to environmental stressors and pathogens.

Our study provides compelling evidence for the potential of ACCA as a bioactive compound, underscored by its strong binding affinity and stable interaction with the 7Q3S protein. However, we acknowledge the need for extensive research to understand the molecular pathways through which ACCA enhances plant resilience. This knowledge could expedite the formulation of targeted strategies for crop protection and enhancement. Furthermore, our research accentuates the value of computational methodologies in identifying prospective bioactive compounds, an integral step in promoting sustainable agricultural practices and securing food production.

## Conclusion

In the diverse ecosystem of *Zea mays*, various pathogenic microorganisms pose a formidable threat to the plant’s health, significantly impacting the yield and quality of this crucial crop. Furthermore, challenges linked with low-irrigated soils, especially in drought-prone regions with sandy soil composition, pose an enduring hurdle to traditional farming methods. Amid such difficulties, rainfall variability and limited water availability further exacerbate the strain on the crops. In this context, our study reveals a promising avenue to alleviate these issues. We found that the application of compounds derived from both plant and Agrobacteria, especially 1-amino-cyclopropane-1-carboxylic acid (ACCA), could potentially mitigate the negative effects of intermittent soil drying, thereby safeguarding the health and vitality of maize cultivations. ACCA, in particular, displayed remarkable binding energy (−9.98 kcal/mol), highlighting its potential to induce virulence, bolster soil moisture retention, and improve the resilience of *Zea mays* under abiotic stress. More than just a novel discovery, this represents a paradigm shift in our approach to crop management, particularly for maize. By utilizing ACCA during the growth stages of maize plants, we can confer resistance against pathogenic infections and enhance crop yield. This potent compound emerged as a forerunner in our study, demonstrating potential to significantly augment virulence and stress tolerance in maize plants.

Our findings illuminate an exciting prospect for leveraging ACCA’s potential to significantly bolster crop yield, quality, and resilience in *Zea mays*. However, the path forward demands comprehensive exploration. Future research should focus on delving deeper into ACCA’s mechanistic pathway, understanding its optimal concentration for crop application, and exploring potential synergistic effects with other natural chemicals. Furthermore, real-world implementation studies would validate ACCA’s efficacy and practicality under diverse agricultural conditions. Our findings provide a critical stepping stone towards a future of sustainable agriculture, marked by improved crop resilience, high yields, and enhanced food security.

### Limitations of the study

Despite the promising outcomes of this research, it’s crucial to recognize certain limitations that exist in our study and regard them as opportunities for further scientific exploration.

### Computational predictions

While our study relied heavily on computational methodologies such as molecular docking and molecular dynamics simulations, these theoretical models might not completely emulate real-world biological environments. Therefore, translating these findings into practical applications requires careful and rigorous empirical validation.

### Single compound focus

Our study primarily focused on 1-amino-cyclopropane-1-carboxylic acid (ACCA). Though ACCA has shown significant promise, the vast world of natural compounds remains largely unexplored. Future studies could expand upon our research by investigating other potential bioactive agents.

### Limited environmental conditions

Our research predominantly revolved around conditions of drought and pathogenic stress. Other environmental stressors such as salinity, temperature extremes, and nutrient deficiency were not specifically addressed. Hence, subsequent research could seek to understand the role of ACCA under these varied stress conditions.

### Detailed mechanistic pathway

While the study provided insights into the interaction between ACCA and 7Q3S protein, a comprehensive understanding of the exact mechanistic pathway involved in enhanced virulence and stress tolerance is yet to be fully elucidated.

### *In vitro* and *In vivo* validation

The current study was computational in nature. Hence, *in vitro* and *in vivo* experiments are imperative to confirm the effectiveness of ACCA and its impact on maize plant health and yield under practical conditions.

Taken together, these limitations do not diminish the value of our findings, but instead offer a fertile ground for future research and potential breakthroughs. As we continue to refine our understanding of ACCA and its potential applications, we move closer to the goal of promoting sustainable agricultural practices and ensuring food security.

## Data availability statement

The original contributions presented in the study are included in the article/supplementary material, further inquiries can be directed to the corresponding authors.

## Author contributions

SD and AS: conceptualization, investigation, supervision, data curation, formal analysis, resources, writing—original draft, and writing—review and editing. AE, AB, RE, MV, PT, SW and LSW: data analysis, investigation, manuscript preparation, and review. All authors contributed to the article and approved the submitted version.

## Funding

The authors extend their appreciation to the Researchers Supporting Project number (RSP2023R56), King Saud University, Riyadh, Saudi Arabia.

## Conflict of interest

The authors declare that the research was conducted in the absence of any commercial or financial relationships that could be construed as a potential conflict of interest.

## Publisher’s note

All claims expressed in this article are solely those of the authors and do not necessarily represent those of their affiliated organizations, or those of the publisher, the editors and the reviewers. Any product that may be evaluated in this article, or claim that may be made by its manufacturer, is not guaranteed or endorsed by the publisher.

## Footnotes

^1^ https://www.rcsb.org/7Q3S
